# Integrating multi-omics and machine learning survival frameworks to build a prognostic model based on immune function and cell death patterns in a lung adenocarcinoma cohort

**DOI:** 10.3389/fimmu.2024.1460547

**Published:** 2024-09-13

**Authors:** Yiluo Xie, Huili Chen, Mei Tian, Ziqang Wang, Luyao Wang, Jing Zhang, Xiaojing Wang, Chaoqun Lian

**Affiliations:** ^1^ Anhui Province Key Laboratory of Clinical and Preclinical Research in Respiratory Disease, MolecularDiagnosis Center, Joint Research Center for Regional Diseases of Institute of Health and Medicine (IHM), First Affiliated Hospital of Bengbu Medical University, Bengbu, China; ^2^ Department of Clinical Medicine, Bengbu Medical University, Bengbu, China; ^3^ Research Center of Clinical Laboratory Science, Bengbu Medical University, Bengbu, China; ^4^ Department of Genetics, School of Life Sciences, Bengbu Medical University, Bengbu, China

**Keywords:** lung adenocarcinoma, precision medicine, machine learning, programmed cell death, immunotherapy efficacy

## Abstract

**Introduction:**

The programmed cell death (PCD) plays a key role in the development and progression of lung adenocarcinoma. In addition, immune-related genes also play a crucial role in cancer progression and patient prognosis. However, further studies are needed to investigate the prognostic significance of the interaction between immune-related genes and cell death in LUAD.

**Methods:**

In this study, 10 clustering algorithms were applied to perform molecular typing based on cell death-related genes, immune-related genes, methylation data and somatic mutation data. And a powerful computational framework was used to investigate the relationship between immune genes and cell death patterns in LUAD patients. A total of 10 commonly used machine learning algorithms were collected and subsequently combined into 101 unique combinations, and we constructed an immune-associated programmed cell death model (PIGRS) using the machine learning model that exhibited the best performance. Finally, based on a series of in vitro experiments used to explore the role of PSME3 in LUAD.

**Results:**

We used 10 clustering algorithms and multi-omics data to categorize TCGA-LUAD patients into three subtypes. patients with the CS3 subtype had the best prognosis, whereas patients with the CS1 and CS2 subtypes had a poorer prognosis. PIGRS, a combination of 15 high-impact genes, showed strong prognostic performance for LUAD patients. PIGRS has a very strong prognostic efficacy compared to our collection. In conclusion, we found that PSME3 has been little studied in lung adenocarcinoma and may be a novel prognostic factor in lung adenocarcinoma.

**Discussion:**

Three LUAD subtypes with different molecular features and clinical significance were successfully identified by bioinformatic analysis, and PIGRS was constructed using a powerful machine learning framework. and investigated PSME3, which may affect apoptosis in lung adenocarcinoma cells through the PI3K/AKT/Bcl-2 signaling pathway.

## Introduction

Lung cancer is by far the most devastating and prevalent malignancy with the highest cancer-related mortality rate and is the leading cause of tumor-related deaths (1, 2). Of these, lung adenocarcinoma (LUAD) is the most common histologic subtype (3, 4). Lung adenocarcinoma (LUAD) is the most common pathologic subtype of lung cancer, accounting for approximately 40% of all lung cancer cases (5). Treatment modalities for LUAD include a variety of approaches, including cell death therapy, gene therapy, immunotherapy, conventional radiotherapy and chemotherapy (6, 7). Despite significant advances in combination therapy strategies for LUAD, the average 5-year survival rate for LUAD is approximately 15% (8). This calls for a search for effective combination therapy strategies for the treatment of LUAD.

Programmed cell death (PCD), also known as regulatory cell death, plays a key role in maintaining tissue homeostasis and eliminating damaged or unwanted cells (9). PCD consists of ferroptosis, apoptosis, pyroptosis, autophagy, necroptosis, cuproptosis, parthanatos, entotic cell death, netotic cell death, lysosome-dependent cell death, alkaliptosis, oxeiptosis, netosis, immunogenic, anoikis, paraptosis, methuosis, entosis, mpt-driven necrosis and disulfidptosis (9–11). Apoptosis is the process by which the body removes injured or unwanted cells and plays a crucial role in various physiological processes. The most critical feature of Necroptosis is the formation of necrosomes, which is a multi-step process (12, 13). Entotic cell death is induced by actinomyosin-dependent cellular internalization (entosis) and is executed through lysosomal degradation (10, 14). Pyroptosis, characterized by cell swelling, lysis, and release of large amounts of pro-inflammatory factors, is a type of inflammation that regulates cell death (15). Parthanatos is a distinct and highly programmed cell death that occurs through over-excitation of the nuclease PARP-1 (16). In addition, the role of other forms of PCD, such as ferroptosis, cuproptosis, and disulfidptosis in LUAD has been widely discussed (17–20). However, the study of these forms of PCD in LUAD remains unclear.

In order to stop lung adenocarcinoma cells from avoiding death and proliferating, a thorough comprehension of these cell death patterns is crucial for creating more efficacious therapies. Explaining various death patterns offers vital understanding into the underlying processes of lung adenocarcinoma. Describing diverse patterns of cell death, their reactions to immunotherapy, alterations in the tumor’s microenvironment, and their predictive value for lung adenocarcinoma patients could provide fresh insights into the emergence and development of lung adenocarcinoma and the prospects of immunotherapy. In this study, we integrated transcriptomic, epigenetic, and somatic mutation data from LUAD patients, used 10 clustering algorithms to identify the three subtypes, and analyzed the differences between the subtypes to characterize key events in the development of LUAD. In addition, we successfully constructed a model with strong prognostic efficacy (PIGRS) using 10 machine learning, and 101 combined machine learning algorithms. This study informs precision medicine for LUAD patients.

## Methods

### Data collection

Clinical information, transcriptome expression, somatic mutations, and copy number variation (CNA) and DNA methylation data for LUAD patients were obtained from the TCGA website (https://portal.gdc.cancer.gov/) (21). After integrating gene expression, methylation, mutation, and copy number variation data from LUAD patients, a final multi-omics dataset of 418 patients was selected for subsequent analysis. In addition, we obtained clinicopathologic information and genome-wide expression data from the Gene Expression Omnibus (GEO) database for two other LUAD cohorts, GSE31210 (n=226) and GSE50081 (n=127), as well as immunotherapy cohorts (GSE91061 and GSE78220) (22–25). From http://researchpub.gene.com/IMvigor210CoreBiologies IMvigor210 dataset (26). To improve comparability between datasets, all RNA-seq data were converted to transcripts per million (TPM) format and corrected for batch effects using the "combat" function of the "sva" package. All data were log-transformed prior to analysis. Single-cell data were processed as in our previous analysis (27). TIDE scores for LUAD patients predicting ICB response were calculated on the TIDE website (http://tide.dfci.harvard.edu) (28).

### Identification of immune-related and PCD-related genes

From IMMPORT (29), we extracted immune-related genes (IRGs) (**Supplementary File S1**). We collected 20 PCD patterns and key regulatory genes from the literature, including 14 genes related to disulfidptosis and 39 genes related to MPT-driven necrosis, etc (20, 30, 31) (**Supplementary File S2**).

### Identification of molecular subtypes

In this study, we used the R package “MOVICS” to establish a new classification of LUAD based on multi-omics data of PCD.mRNA expression, IRG.mRNA expression, DNA methylation, and somatic mutation data in a similar way as Chu.et.al (32, 33). We analyzed the clustering prediction index (CPI). We analyzed the clustering prediction index (CPI) and the disparity statistic to determine the optimal number of cancer subtypes (34). We used 10 clustering algorithms (CIMLR, ConsensusClustering, SNF, iClusterBayes, PINSPlus, moCluster, NEMO, IntNMF, COCA, and LRA) to categorize the multi-omics data, and after integrating the results of the 10 clusters, we ultimately decided to classify LUAD patients into three types.

### Identification of different molecular signatures

In order to reveal the underlying biology, the 3 subtypes we established were characterized by gene set variation analysis (GSVA) using GO terms (35). The first 10 GO pathways and the network of relationships between the subtypes were visualized. We evaluated the infiltration of the 3 subtypes using the CIBERSORT algorithm (36). Heatmaps revealed differences in immune cell infiltration. In addition, differences in the expression levels of immune checkpoints between the three subtypes were analyzed, further revealing the link between subtype and immunity. In addition, we used the built-in functionality of the MOVICS package to calculate the fraction of the genome altered by copy number amplification or deletion (FGA) (37). In addition, we also calculated the TMB for each subtype.

### Identification of different subtypes of drug sensitivity

To evaluate the response of different subtypes to the drugs, human cancer cell lines (CCLE) from GDSC (https://www.cancerrxgene.org/) were used as a training cohort and the R package “pRRohetic” was used to predict the corresponding sensitivities (38). The half maximal inhibitory concentration (IC50) was calculated by ridge regression and set as a metric to compare different agents. We used three common drugs (“Cisplatin”, “Paclitaxel”, “Sorafenib”) to measure the predictive effect. To validate the accuracy of our molecular classification prediction predictions, we utilize the “combat” function in the sva package to remove the batch effects in the GSE50081 and GSE31210 datasets, and integrate them into a unified dataset, called META, which serves as an external dataset for validation. We used the NTP algorithm to molecularly classify the external dataset and calculate the difference in prognosis between the different classifications (39).

### Constructing prognostic models

We use 10 machine learning algorithms for a total of 101 machine learning combinations (40, 41). The 10 machine learning are SVM, Lasso, GBM, RSF Enet, Stepwise Cox, Ridge, CoxBoost, SuperPC, and PplsRcox. we constructed the immune-associated programmed cell death model (PIGRS) by Lasso + GBM. We ranked each model according to its C-index and defined the model with the highest C-index as the best model, as in our previous strategy, and a detailed description of each algorithm and the specific implementation of the various combinations can be found in a previous study (25). To confirm the predictive utility of PIGRS, we calculated the area under the receiver operating characteristic curve (AUC) using the timeROC software package (42). In addition, we collected model indices from previous researchers and compared the PIGRS with the previous models, which showed that our PIGRS has strong prognostic efficacy.

### Pathway enrichment analysis

In order to investigate the genes that showed significant differential expression between the high and low PIGRS groups, this study used “limma” to analyze the differences between the two groups (FDR < 0.05 and log2 fold change (FC) > 1). We used the “clusterProfiler” package for gene set enrichment analysis (GSEA) and Kyoto Encyclopedia of Genes and Genomes (KEGG) analysis (43). In addition, we also used the “GSVA” package to perform gene set variation analysis (GSVA) to further reveal the underlying mechanisms among different subgroups (35).

### Analysis of genomic variation among PIGRS risk subgroups

Mutation with built-in tumor heterogeneity (MATH) is a method to quantify intra-tumor heterogeneity (ITH) based on the distribution of mutant alleles. The prognostic significance of MATH has been investigated in a wide variety of tumors, including head and neck, colorectal, and breast cancers (44–47). In this study, we calculated the MATH score for each LUAD patient according to the previously described method and performed survival analysis based on their MATH score (48). We utilized the R package “maftools” to study somatic mutations associated with PIGRS and generated waterfall plots showing mutations in LUAD patients in the high and low PIGRS groups. In addition, we calculated the Tumor Mutational Burden (TMB) score for each LUAD patient and explored the relationship between high and low PIGRS groups and TMB and survival analysis.

### Cell culture and transfection

Lung adenocarcinoma cell lines A549 and H1299 were mainly purchased from the cell bank of the Chinese Academy of Sciences (Shanghai, China). We used A549 and H1299 cells for *in vitro* culture experiments, cultured in DMEM medium and RPMI 1640 medium (Gibco, ThermoFisher Scientific, United States) supplemented with 10% fetal bovine serum, 1% penicillin and streptomycin (Gibco). Small interfering RNA (siRNA) targeting (proteasome activator subunit 3) PSME3 and interfering RNA control were purchased from Gemma Genetics (Shanghai, China). For transient transfection, A549 and H1299 cells were transfected with siRNA using transfection reagent (Lipofectamine 2000) for 12h, followed by functional assays and subsequent experiments. siRNA sequence and primer sequence (**Supplementary Table 3**).

### Cell proliferation

Cell proliferation and colony formation assay assay A549 and H1299 cells were cultured in 96-well plates (3,000 cells/well) 24 hours after transfection with PSME3 siRNA. The proliferative capacity of the treated cells was assayed at 4, 24, 48 and 72 hours. 10% Cell Counting Kit-8 (CCK8) reagent (Bio-sharp, Hefei, China) was added to each plate according to the kit instructions, and the OD450 values were analyzed by an enzyme marker (BioTek, United States). Regarding clonal spot experiments, 2000 cells were inoculated in cell culture plates and allowed to grow until visible colonies were formed. Then we fixed the clones with paraformaldehyde for 15 min, stained the clones with 1% crystal violet for 20 min, and counted the number of clones (>50 cells).

### Transwell detects cell migration and invasion

Transwell migration and wound healing assay A549 and H1299 cells were transfected with PSME3 siRNA for 24 h and cultured in 24-well culture plates with 8 mm pore membrane inserts to measure cell migration capacity. 4 × 10^4^ cells were inoculated in the upper chamber of a transwell with 200 ul of serum-free medium, and 800 μl of medium containing 10% FBS was added to the lower chamber. After 48 h of incubation, cells migrating across the membrane were fixed with 4% paraformaldehyde, stained with 1% crystal violet and counted under a light microscope (50×).

### RT-qPCR and western blot analysis

Total RNA was extracted using TRIzol reagent (Invitrogen). The SYBR Green qPCR blend (Vazyme, China) facilitated the synthesis of cDNA for immediate PCR analysis. Dissolve protein samples in Lithium Dodecyl Sulfate (LDS) Sample Buffer (Invitrogen). Aliquots of the total protein extract are separated on 10% SDS-PAGE gels (30 min at 90 V and 90 min at 120 V) and transferred to a polyvinylidene fluoride membrane. The transfer was performed at 100 V for 2 hours using a Bio-Rad transfer device. The membrane is then closed in 5% BSA solution for 1 hour at room temperature. The appropriate primary antibodies are incubated at 4°C overnight. Primary antibodies were as follows: Akt and p-Akt (Ser473) (Cell Signaling Technology, USA: 1:1000); β-actain and PSME3 (Santa Cruz, USA: 1:1000).

### Statistical analysis

All statistical analyses were performed using R software (version 4.1.0). Wilcoxon test was used for comparing two groups, while Spearman or Pearson correlation was used for correlation matrices. The Log-rank test was used to The Log-rank test was used to assess survival differences through K-M curves, where statistical significance was defined as p-value < 0.05. The Log-rank test was also used to assess survival differences through K-M curves.

## Results

### Identification of three molecular subtypes based on the TCGA-LUAD cohort

**Figure 1:**
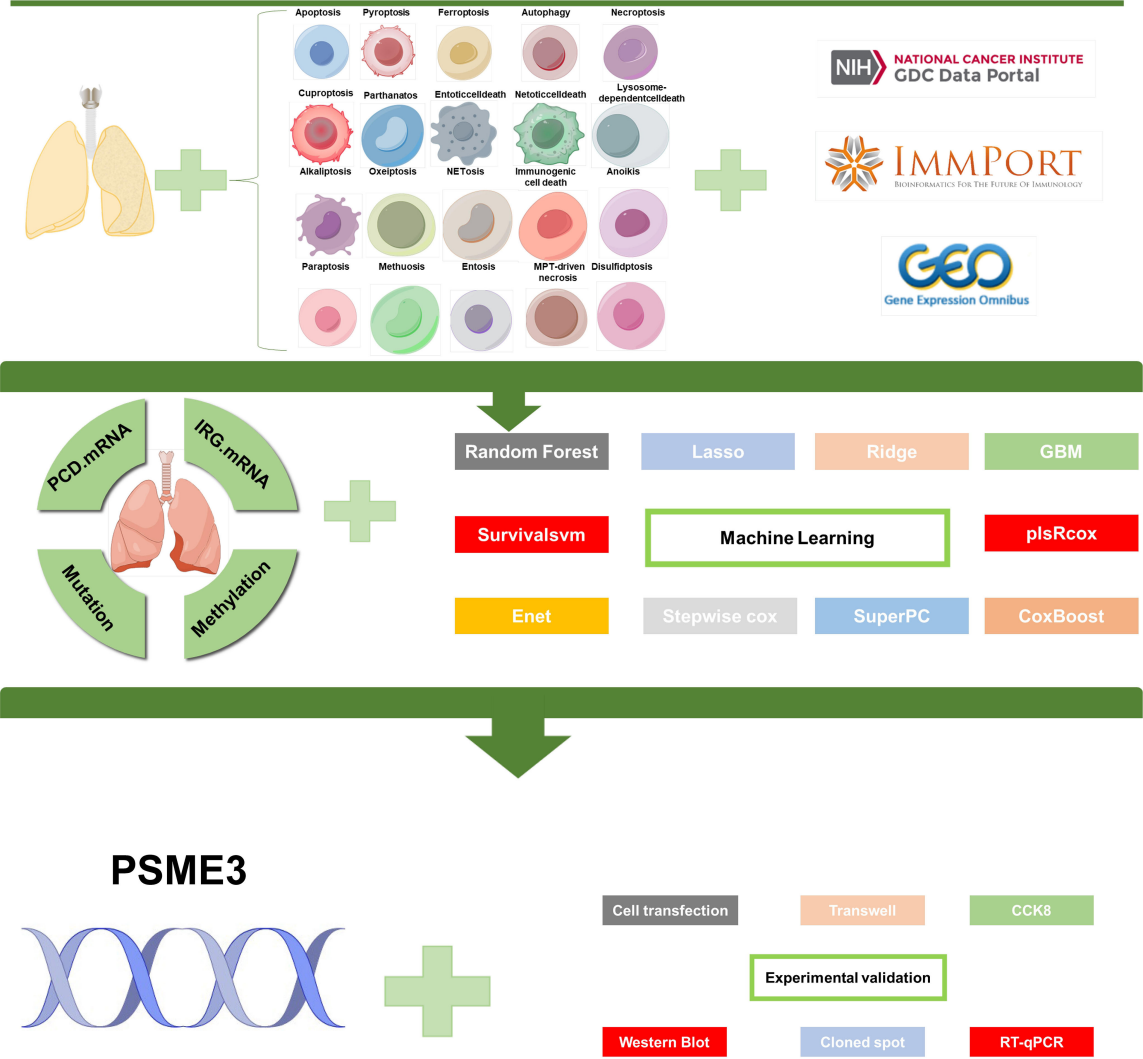
Flowchart of this study.

**Figure 2:**
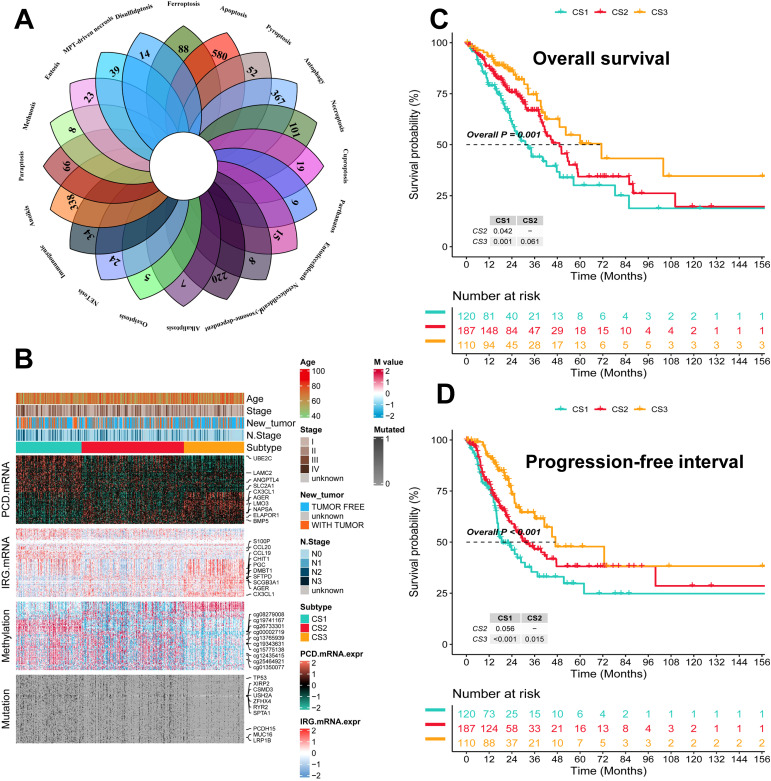
Identification of three subtypes and assessment of clinical prognosis of different subtypes. **(A)** Veen plot demonstrating 20 cell death genes; **(B)** multi-omics characterization of the three subtypes; **(C)** OS survival prognosis of the three subtypes; **(D)** PFI prognosis of the three subtypes.

The flowchart of this study is shown in **Figure 1**. We collected genes related to cell death from the literature(**Figure 2A**), and subsequently, we included immune-related genes in our analysis. After matching cell death-related genes, immune-related genes, methylation, and mutation data, 417 TCGA-LUAD samples were included in the subsequent analysis. The number of clusters was estimated using the clustering prediction index (CPI) and gap statistics (**Supplementary Figure 1A**). Ten conventional algorithms in the R package “MOVICS” were used to categorize the patients into three predefined multi-omics subtypes (CS), which were ultimately consolidated into robust classifications by integrating consensus. The results of the contour analysis also illustrate the moderate similarity of the samples in each cluster, with contour scores of 0.85, 0.48, and 0.77 for CS1, CS2, and CS3, respectively (**Supplementary Figure 1C**). Based on the ten algorithms, LUAD patients were categorized into three subtypes: the CS1, CS2 and CS3 (**Figure 2B**). Survival analyses showed a significant difference in CS3 prognosis among these three subtypes in overall survival and progression-free period (P = 0.001; **Figures 2C, D**). CS3 patients had a relatively better prognosis, whereas CS1 patients had the worst prognosis.

### Assessing the characteristics of different subtypes

**Figure 3:**
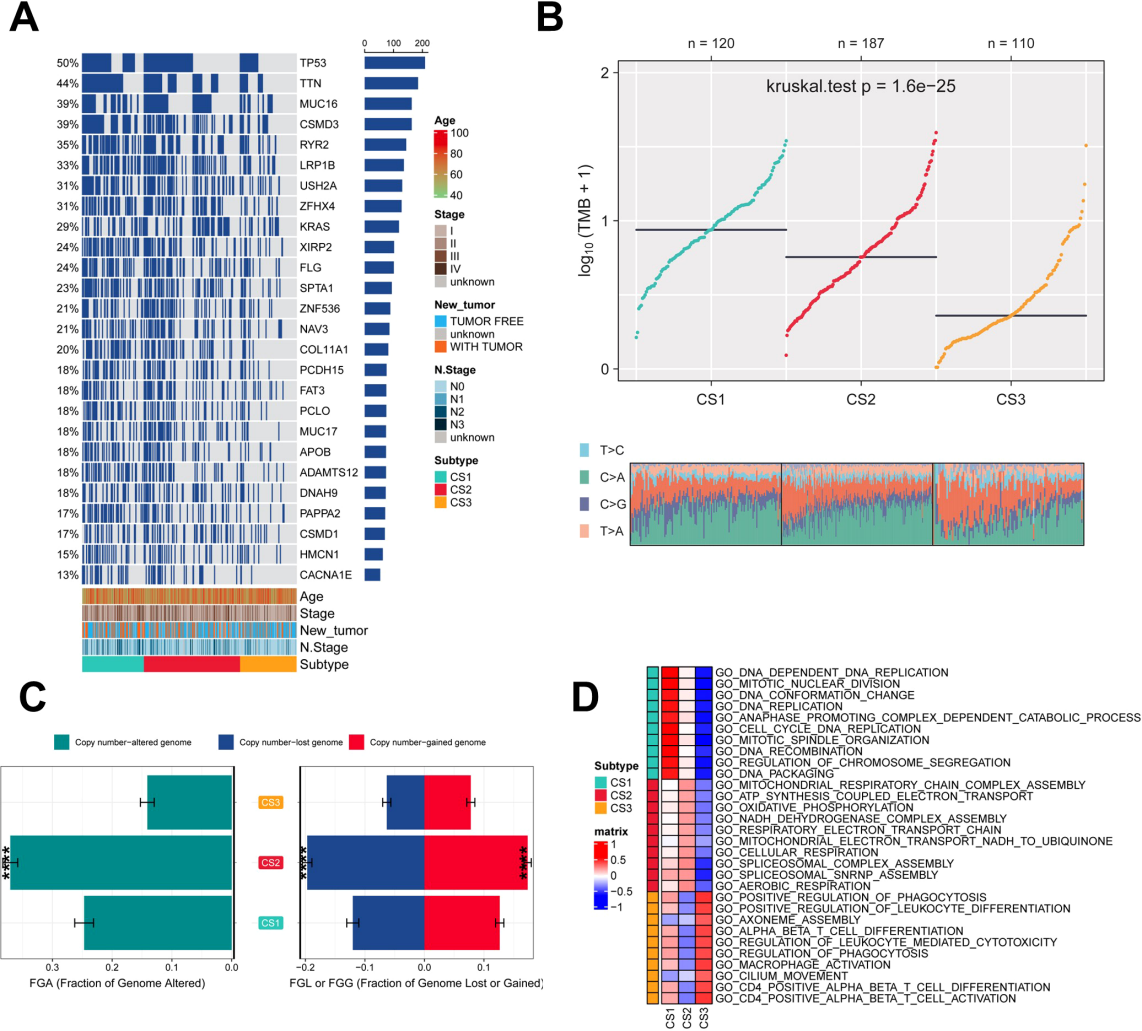
Assessment of mutation status and pathway analysis across different subtypes. **(A)** Veen plot demonstrating 20 cell death genes; **(B)** multi-omics characterization of the three subtypes; **(C)** OS survival prognosis of the three subtypes; **(D)** PFI prognosis of the three subtypes.

Genetic mutations and CNV play key roles in tumor development and progression (49). Therefore, the present study compared the genetic alterations between the different subtypes. First, we compared the differences in individual gene mutations among the three subtypes. TTN, TP53, MUC16, CSMD3, and RYR2 were the five genes with the highest mutation frequencies in LUAD patients. Mutation frequencies were higher in the CS1 and CS2 groups compared to the CS3 group (**Figure 3A**). Tumor mutation Burden (TMB) is widely used in clinical practice (50, 51). The CS1 group had the highest TMB, while the CS3 group had the lowest TMB (**Figure 3B**). We also assessed the three subtypes of CNV by calculating the FGA score to investigate chromosomal instability. We found that CS3 had better chromosomal stability and significantly lower copy number loss or gain compared to the other subtypes (**Figure 3C**). To further understand the differences between the three subtypes, we performed gene ontology (GO) terminology pathway enrichment to identify subtype-specific activated signaling pathways. We observed the presence of DNA_DEPENDENT_DNA_REPLICATION, CELL_CYCLE_DNA_REPLICATION, and DNA_REPLICATION in patients with CS1. As for MoS2, ATP_SYNTHESIS_COUPLED_ELECTRON_ TRANSPORT, MITOCHONDRIAL_RESPIRATORY_CHAIN_COMPLEX_ASSEMBLY and NADH_DEHYDROGENASE_COMPLEX_ASSEMBLY were enriched. Meanwhile, CS3 was significantly enriched for the activation of immune-related pathways, including CD4_POSITIVE_ALPHA_BETA_T_CELL_ACTIVATION, CD4_POSITIVE_ALPHA_BETA_T_CELL_DIFFERENTIATION, and MACROPHAGE_ ACTIVATION (**Figure 3D**).

### Immune infiltration of different subtypes

**Figure 4:**
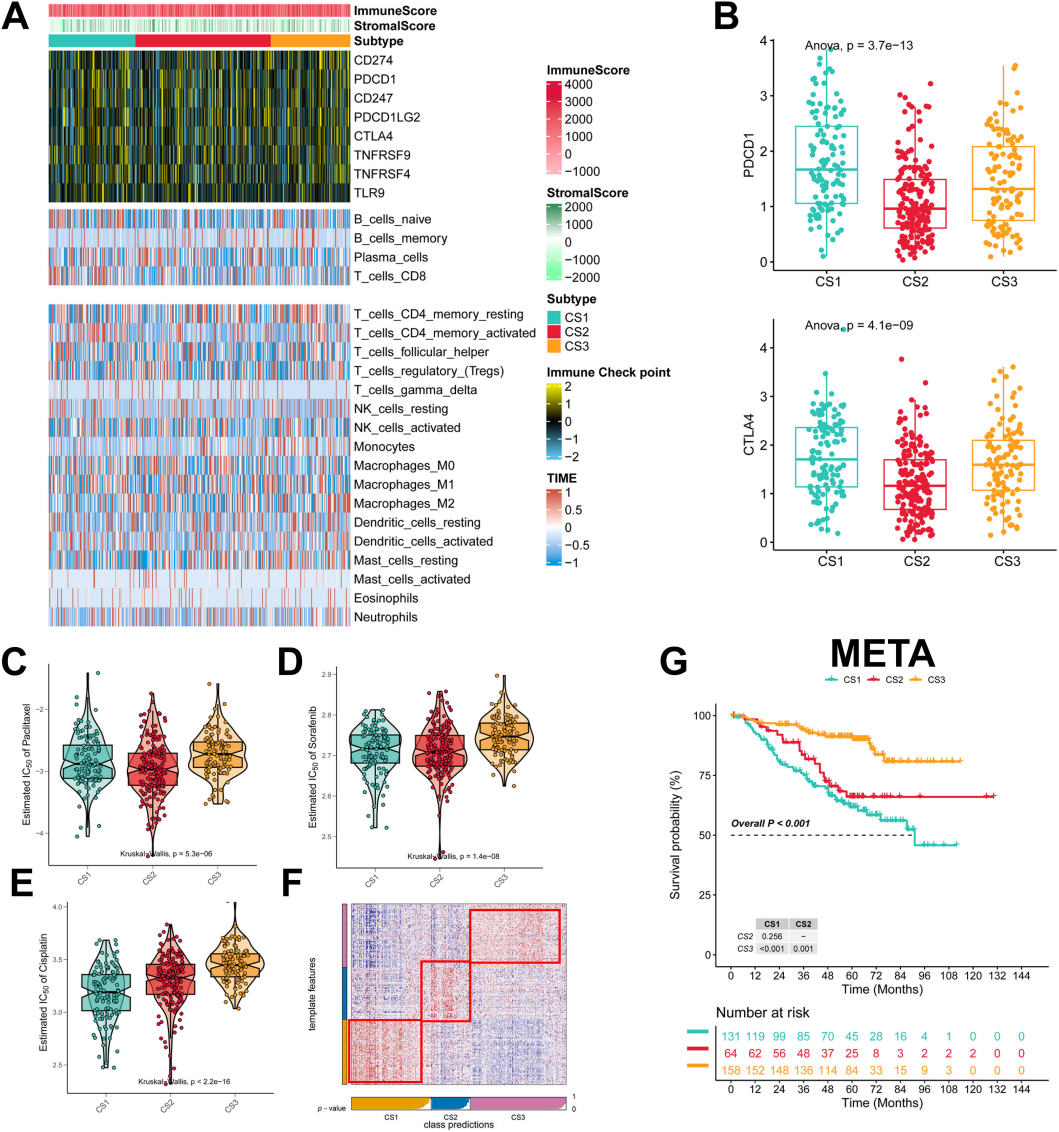
Assessment of the immune microenvironment in different subtypes. **(A)** Heatmap showing the expression of immune checkpoint genes and the level of tumor-infiltrating lymphocytes in each subtype; **(B)** Expression levels of CTLA4 and PDCD1; **(C-E)** IC50 values of commonly used chemotherapeutic agents; **(F)** Heatmap of NTPs generated according to subtype-specific up-regulation of biomarkers identified in the LUAD cohort; **(G)** km survival curves of the META cohort.

To explore the immune infiltration of the three subtypes, we investigated the distribution of immune cells and the abundance of immune checkpoint expression in the three subtypes. patients in the CS3 group had the highest levels of immune checkpoint expression and immune cell infiltration, whereas patients in the CS1 group had the lowest levels of immune checkpoint expression and immune cell infiltration (**Figure 4A**; **Supplementary Figures 2A–C**). As for the mRNA levels of immune checkpoints, we found that CS1 contained higher CTLA4 levels and the highest PD-1 levels (**Figure 4B**). We also analyzed the drug sensitivity of the three subtypes to cisplatin, paclitaxel, and docetaxel. patients in the CS2 cohort were most sensitive to sorafenib and paclitaxel, whereas those in the CS1 cohort had the highest sensitivity to cisplatin (**Figures 4C–E**). In addition we collected results on mRNA expression profiles and response to anti-PD-1 therapy in the 3 cohorts. Patients in the 3 cohorts were first categorized into three CSs, with CS3 resulting in better clinical therapeutic benefit (**Supplementary Figures 3A–C**). We identified the top 200 up-regulated biomarkers for the three subtypes using “limma” (P < 0.05, **Supplementary Figure 1D**). Subsequently, we selected the META dataset (containing GSE31210 and GSE50081). The NTP method was used to predict the prognosis of each dataset based on the specific upregulation of biomarkers in each subtype (**Figure 4F**). As shown, the NTP results indicated that CS3 had the best prognosis in all externally validated datasets, whereas CS1 and CS2 had a poorer prognosis, which was consistent with the initial subtype prediction (**Figure 4G**). These results indicate that our subtyping method is reliable.

### Machine learning to build PIGRS

**Figure 5:**
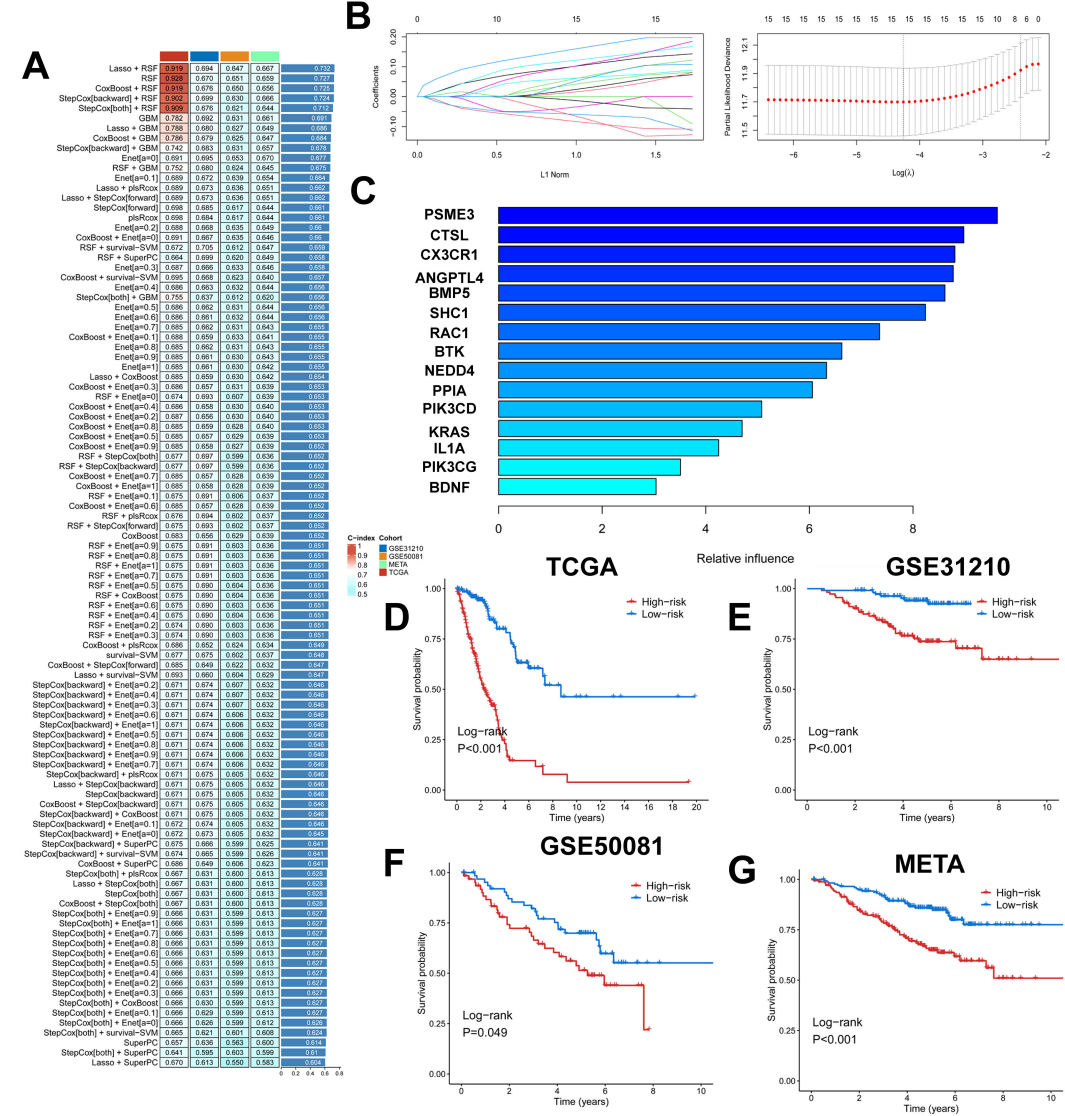
Multiple machine learning constructs for PIGRS. **(A)** Heatmap showing 101 machine learning; **(B)** Lasso screening genes; **(C)** GBM algorithm showing the importance of different genes; **(D-G)** km survival curves for high and low PIGRS **(D)** TCGA; **(E)** GSE31210; **(F)** GSE50081; **(G)** META cohort.

We took the intersection of cell death genes and immune-related genes to obtain 31 genes (**Supplementary Figure 4A**). Based on the 31 prognostically relevant immune and cell death genes (PIRGs), this study used machine learning to construct prognostic models from these genes. Using the TCGA dataset, we constructed 101 prognostic models and evaluated the performance of each model in three independent validation sets-GSE31210, GSE50081, and META. We note that the “Lasso + RSF”, “RSF”, “CoxBoost + RSF”, “Stepcox [backward] + RSF”, and “Stepcox [both] + RSF” models have very high C-Indexes in the TCGA dataset, but their performances degrade in the remaining three validation sets. This suggests an overfitting phenomenon. However, the GBM model contains a total of 31 genes, whereas the Lasso + GBM model contains only 15 genes but achieves comparable prediction, and in order to ensure that the models have consistently strong predictive power across all four datasets, we chose the “Lasso + GBM” combination, which creates a model with an average C-Index of 0.686 across the four datasets (**Figure 5A**). The Lasso algorithm selected 15 PIRGs (**Figure 5B**), and the GBM algorithm assessed their relative impact in the model (**Figure 5C**). Finally, we derived a Lasso + GBM model consisting of 15 PIRGs. Kaplan-Meier analysis showed that all 15 PIRGs had a significant impact on the prognosis of LUAD patients (**Supplementary Figure 4B**). Using the expression of these 15 PIRGs for each patient and weighting them according to their relative influence, the model calculated a risk score for each individual. We named this risk model the cell death, immune-related gene model (PIGRS).

The median PIGRS was the dividing line that classified patients into two different groups. Patients in the high PIGRS group had a significantly worse prognosis compared to the low PIGRS group, not only in the training set TCGA (**Figure 5D**), but also in the two external validation cohorts, i.e., the GSE31210 (**Figure 5E**), GSE50081 (**Figure 5F**), and META (**Figure 5G**) cohorts. The clinical trilinear table can be found in **Table 1**.

**Table 1:** TCGA-LUAD Clinical characteristics.

Characteristics	High (N=208)	Low (N=209)	P-value
Gender			
male	105 (50.5%)	85 (40.7%)	**0.056**
female	103 (49.5%)	124 (59.3%)	
Stage			
I	90 (43.3%)	137 (65.6%)	**5.7e-05**
II	62 (29.8%)	39 (18.7%)	
III	45 (21.6%)	21 (10.0%)	
IV	10 (4.8%)	9 (4.3%)	
unknown	1 (0.5%)	3 (1.4%)	
T stage			
T1	58 (27.9%)	88 (42.1%)	**0.022**
T2	117 (56.3%)	100 (47.8%)	
T3	24 (11.5%)	12 (5.7%)	
T4	8 (3.8%)	8 (3.8%)	
TX	1 (0.5%)	1 (0.5%)	
N stage			
N0	118 (56.7%)	157 (75.1%)	**2.5e-05**
N1	46 (22.1%)	29 (13.9%)	
N2	42 (20.2%)	16 (7.7%)	
N3	1 (0.5%)	0 (0%)	
unknown	1 (0.5%)	7 (3.3%)	
M stage			
M0	140 (67.3%)	120 (57.4%)	**0.078**
M1	10 (4.8%)	9 (4.3%)	
unknown	58 (27.9%)	80 (38.3%)	

Bold values represent P-values.

### Comparison of PIGRS with other models

**Figure 6:**
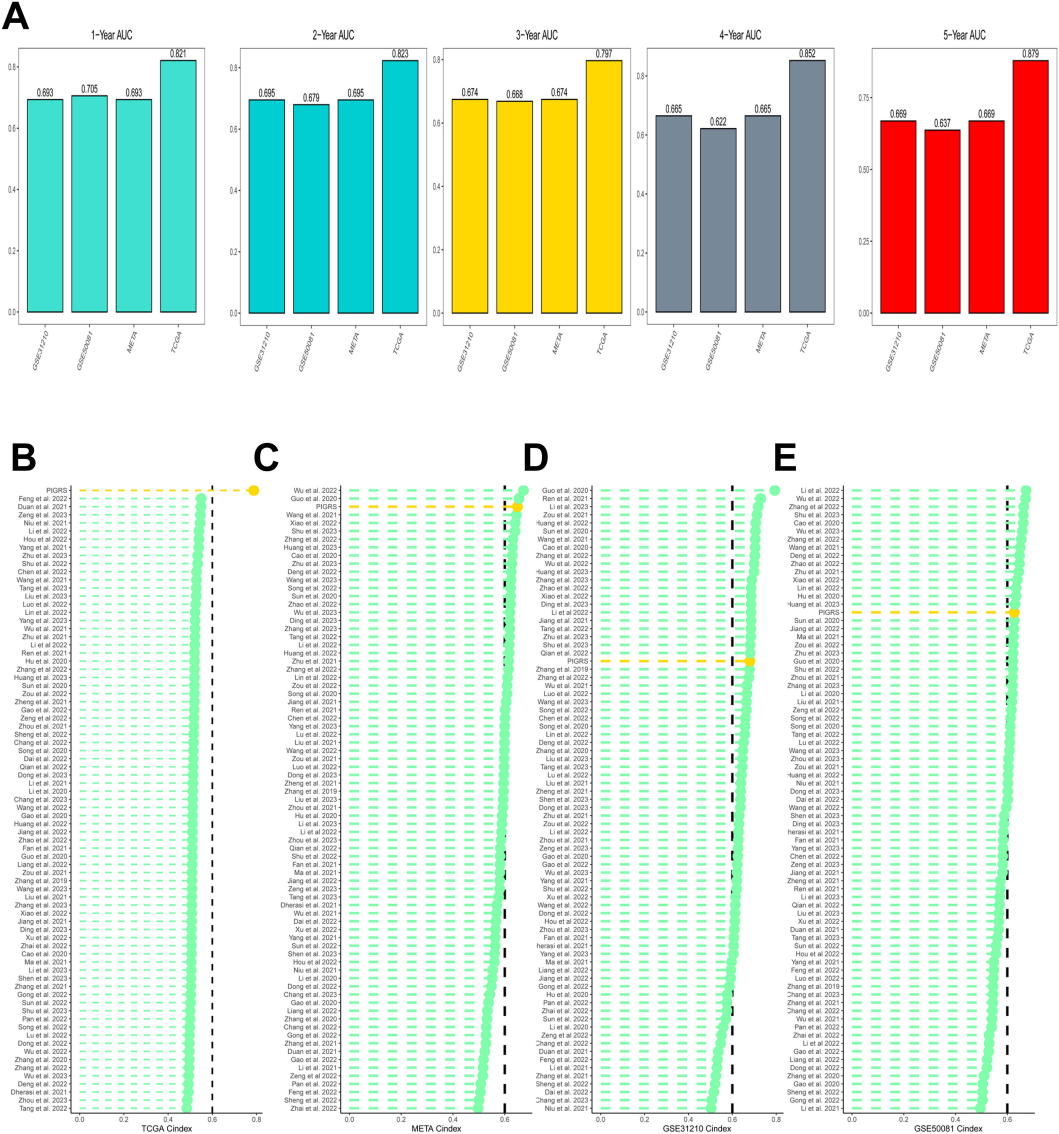
Model comparisons. **(A)** Evaluation of PIGRS for predicting 1-, 2-, 3-, 4-, and 5-year survivability in patients; **(B-E)** PIGRS compared with published models.

Based on the ROC analysis, PIGRS was able to distinguish well between one-, two-, three-, four-, and five-year AUCs of 0.821, 0.823, 0.797, 0.852, and 0.879, respectively, in TCGA-LUAD; 0.693, 0.695, 0.674, 0.665, and 0.669 in GSE31210; 0.705, 0.679, 0.668, 0.622, and 0.637 in GSE50081 0.705, 0.679, 0.668, 0.622, and 0.637 in GSE50081; and 0.693, 0.695, 0.674, 0.665, and 0.669 in META (**Figure 6A**). To compare the prognostic efficacy of PIGRS with existing LUAD models, we integrated previous studies that used different biologically significant features, such as m6A (52), copper death (53), necrotic apoptosis (54), Necrotic apoptosis (55), ubiquitin proteasome (56), Autophagy (57) and immune checkpoints (58) etc. Notably, PIGRS exhibited better C-index performance than almost all models in the TCGA-LUAD, GSE31210, GSE50081, and META datasets (**Figures 6B–E**). In conclusion, these findings confirm the idea that PIGRS is a more effective prognostic model for LUAD.

### Pathway enrichment analysis of PIGRS

**Figure 7:**
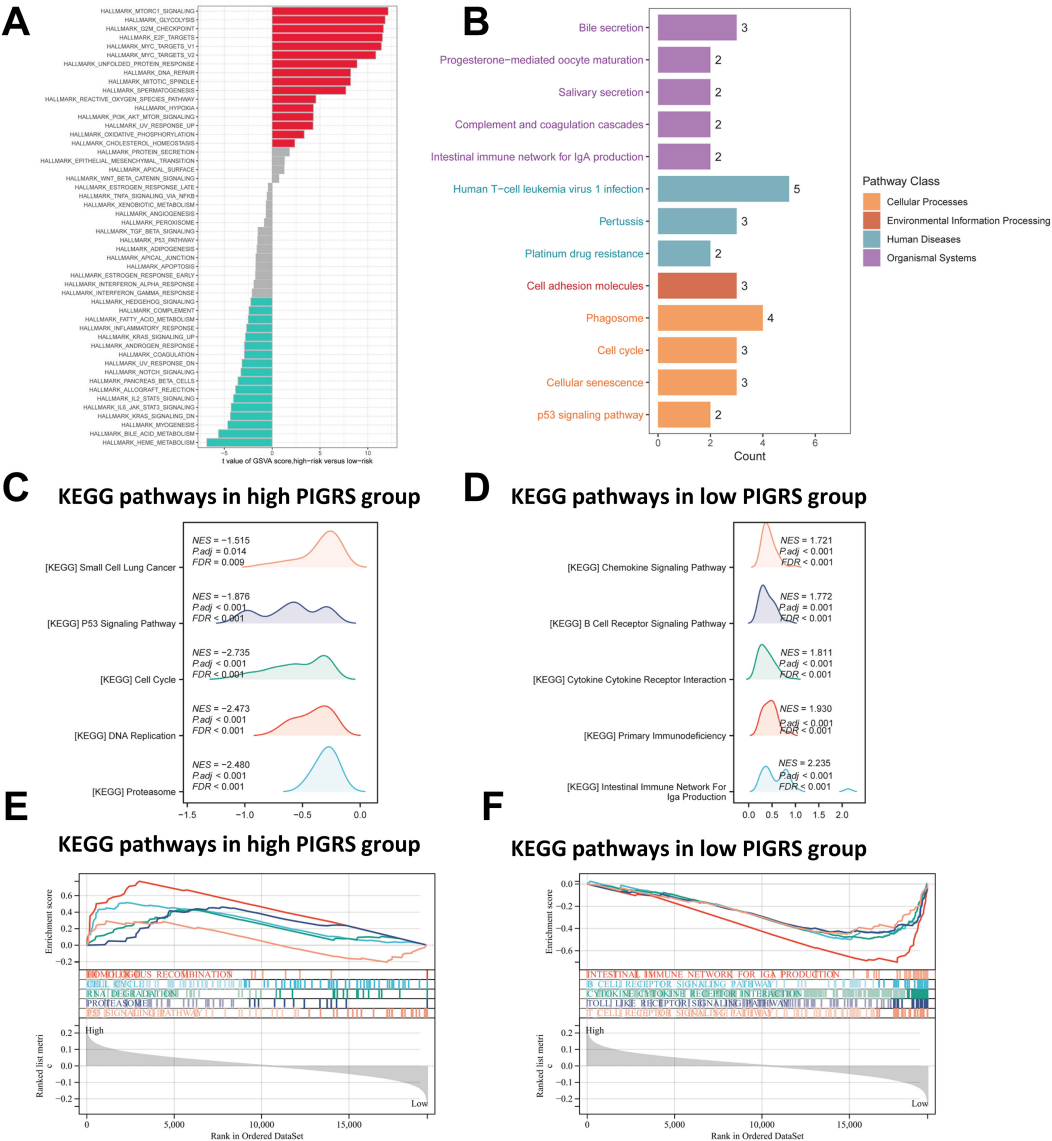
Functional enrichment analysis of the PIGRS group. **(A)** GSVA enrichment analysis in the PIGRS group; **(B)** KEGG enrichment analysis in the PIGRS group; **(C, E)** GSEA enrichment analysis in the high PIGRS group; **(D, F)** GSEA enrichment pathway in the low PIGRS group.

To investigate the biological processes associated with PIGRS in more depth, we performed enrichment analysis. GSVA analyzed the Hallmark pathway that was differentially enriched between the two groups (**Figure 7A**). Kyoto Encyclopedia of Genes and Genomes (KEGG) results are shown in **Figure 7B**. Regarding the organismal system, PIGRS were mainly enriched in Bile secretion, Progesterone-mediated oocyte maturation, and Salivary secretion. In Human Diseases, PIGRS were most concentrated in Human PIGRS are most concentrated in Human T cell leukemia virus 1 infection, Pertussis and Platinum drug resistance. PIGRS are particularly abundant in the Cell adhesion molecules pathway. In Cellular progessing, PIGRS was mainly enriched in Phagosome, Cell cycle, Cellular senescence and p53 signaling pathways. In addition, we utilized GSEA to identify potential pathways associated with PIGRS. In **Figures 7C, E**, the high PIGRS group was significantly enriched in oncogenic-related pathways such as Small Lung cancer, cell cycle, and p53 signaling pathway. In contrast, the low PIGRS group was mainly enriched to some immune-related biological processes, which explained its better prognosis (**Figures 7D, F**).

### Mutational status of PIGRS

**Figure 8:**
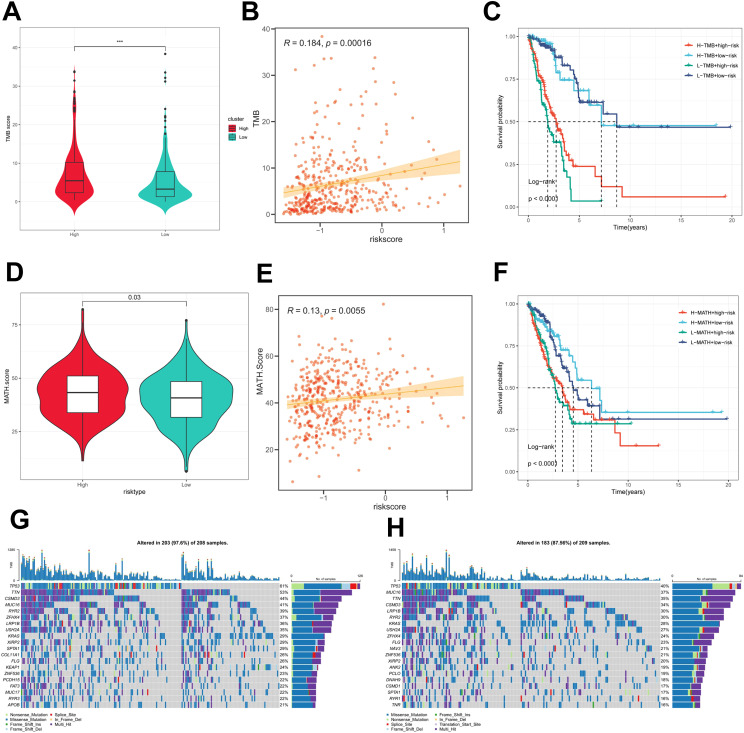
Genomic variation and intra-tumor heterogeneity in different PIGRS subgroups. **(A)** Violin plots demonstrating TMB differences between the high and low PIGRS groups; **(B)** correlation between TMB and PIGRS; **(C)** Kaplan-Meier curves analyzing OS by combining the TMB score and PIGRS risk score. **(D)** Violin plot showing the difference in mutant allele tumor heterogeneity (MATH) scores between the high PIGRS and low PIGRS groups. **(E)** Correlation between MATH and PIGRS; **(F)** Kaplan-Meier curves analyzing OS by combining MATH score and PIGRS risk score. **(G)** Mutation analysis of the high PIGRS group. **(H)** Mutation analysis of the low PIGRS group. ***p < 0.001.

First, there was a significant difference in tumor mutational Burden (TMB) between the high and low PIGRS groups, and the tumor mutational load was significantly higher in the high-risk score group than in the low-risk score group (**Figure 8A**). Then the correlation between PIGRS and TMB was explored, and a Spearman correlation analysis was used, which revealed that there was a significant positive correlation between PIGRS and TMB (R = 0.184, P< 0.001, **Figure 8B**). After integrating the TMB scores, LUAD patients in TCGA were divided into four groups. Survival analysis showed that patients with high TMB and low risk had a significant survival advantage, and the low TMB and high-risk groups exhibited a significant survival disadvantage (**Figure 8C**). Intra-tumor heterogeneity (ITH) is caused by genetic mutations (59). A well-known genomic feature of cancer resulting from the accumulation of mutations.ITH has been shown to be associated with increased malignancy and chemotherapy resistance (60). In this study, we used the Mutant Allele Tumor Heterogeneity (MATH) algorithm to measure ITH in LUAD patients; higher MATH scores were associated with higher ITH. The MATH score was higher in LUAD patients in the high PIGRS group (**Figure 8D**). Then the correlation between PIGRS and MATH was explored, and Spearman correlation analysis was used, and a significant positive correlation was found between PIGRS and MATH (R = 0.13, P< 0.01, **Figure 8E**). We further combined ITH with PIGRS, and we found that patients in the high MATH and low PIGRS groups had a significant survival advantage, and the low MATH and high PIGRS groups showed a significant survival disadvantage suggesting that the combination of these two metrics could better assess the prognosis of LUAD patients (**Figure 8F**). It is well known that genetic mutation is a condition for tumorigenesis. In the TCGA database, we visualized and correlated somatic mutation data based on PIGRS combined with high PIGRS and low PIGRS groups. The three genes with the highest mutation frequencies in the high PIGRS group were TP53 (61%), TTN (53%), and CSMD3 (44%) (**Figures 8G, H**).

### Immunologic properties of PIGRS

**Figure 9:**
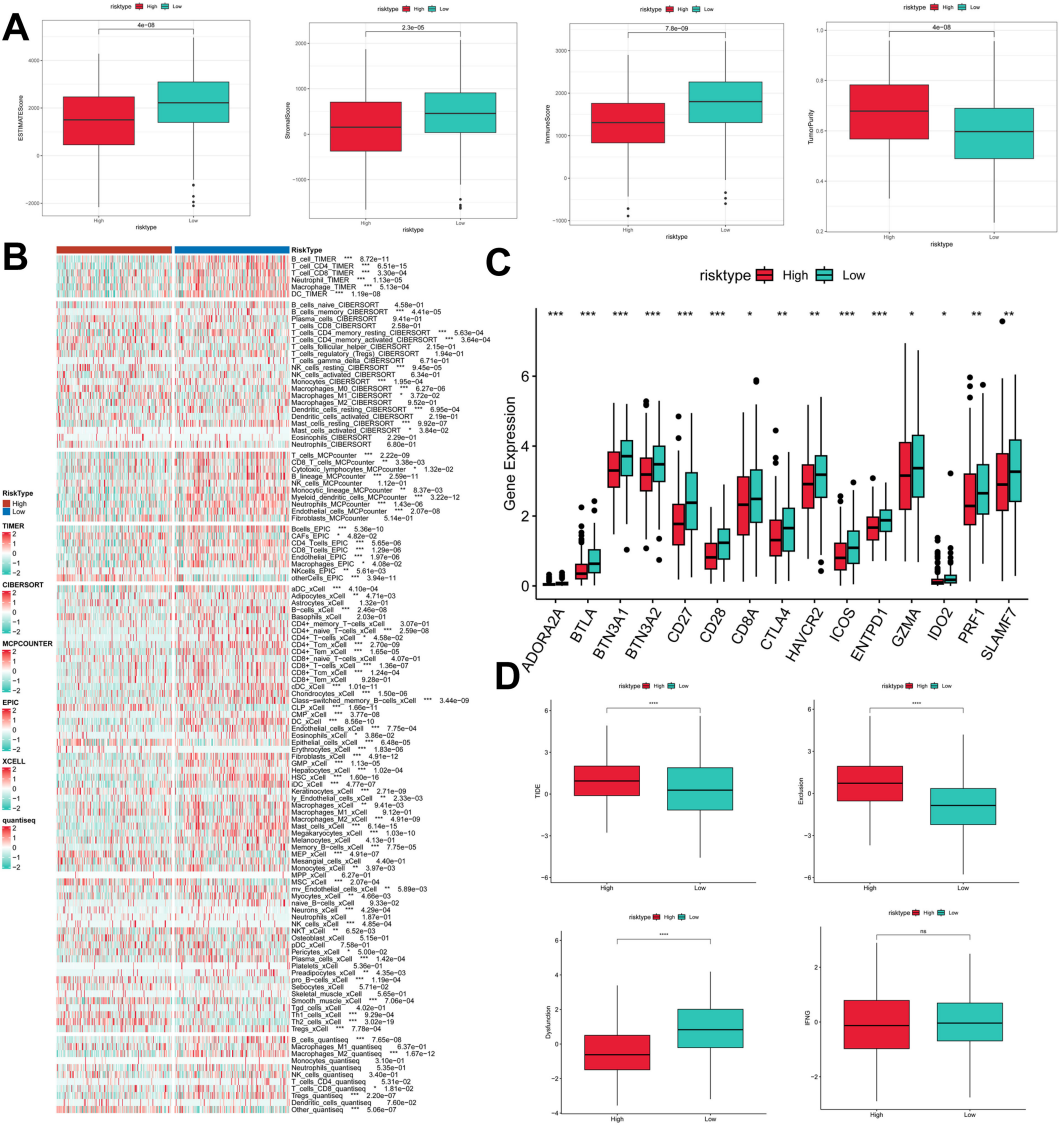
Exploration of the tumor immune microenvironment. **(A)** Stroma score, immunity score, ESTIMATE score, and tumor purity were used to quantify the different immune statuses between the high and low risk groups; **(B)** Heatmap demonstrating the situation of the six immune infiltration algorithms; **(C)** Differential expression of the various immune checkpoints in the high- and low-risk groups; **(D)** Differences in TIDE expression. *p<0.05,**p<0.01,***p < 0.01,****p < 0.0001.

To assess the immune infiltration status of LUAD samples in this study, we used the ESTIMATE algorithm to calculate stromal scores, immune scores, ESTIMATE scores, and tumor purity for the PIGRS risk subgroup. The immunity score and ESTIMATE score were significantly higher in the low PIGRS group, while the tumor purity was higher in the high-risk group (**Figure 9A**). To further analyze the differences in specific immune cell infiltration between the high- and low-PIGRS groups, we quantified the abundance of immune cell infiltration in each sample using six algorithms (TIMER, CIBERSORT, MCPCOUNTER, EPIC, XCELL, quantisep) (**Figure 9B**). The results showed more immune infiltrating cells in the low PIGRS group. Next to explore the differences in immune checkpoint expression between the two subgroups, we examined the expression of immune checkpoints, which showed higher expression in the low PIGRS group (**Figure 9C**). We assessed immune escape between the two groups using TIDE, which showed that the high PIGRS group may have immune escape (**Figure 9D**).

### Immunotherapeutic effects of PIGRS

**Figure 10:**
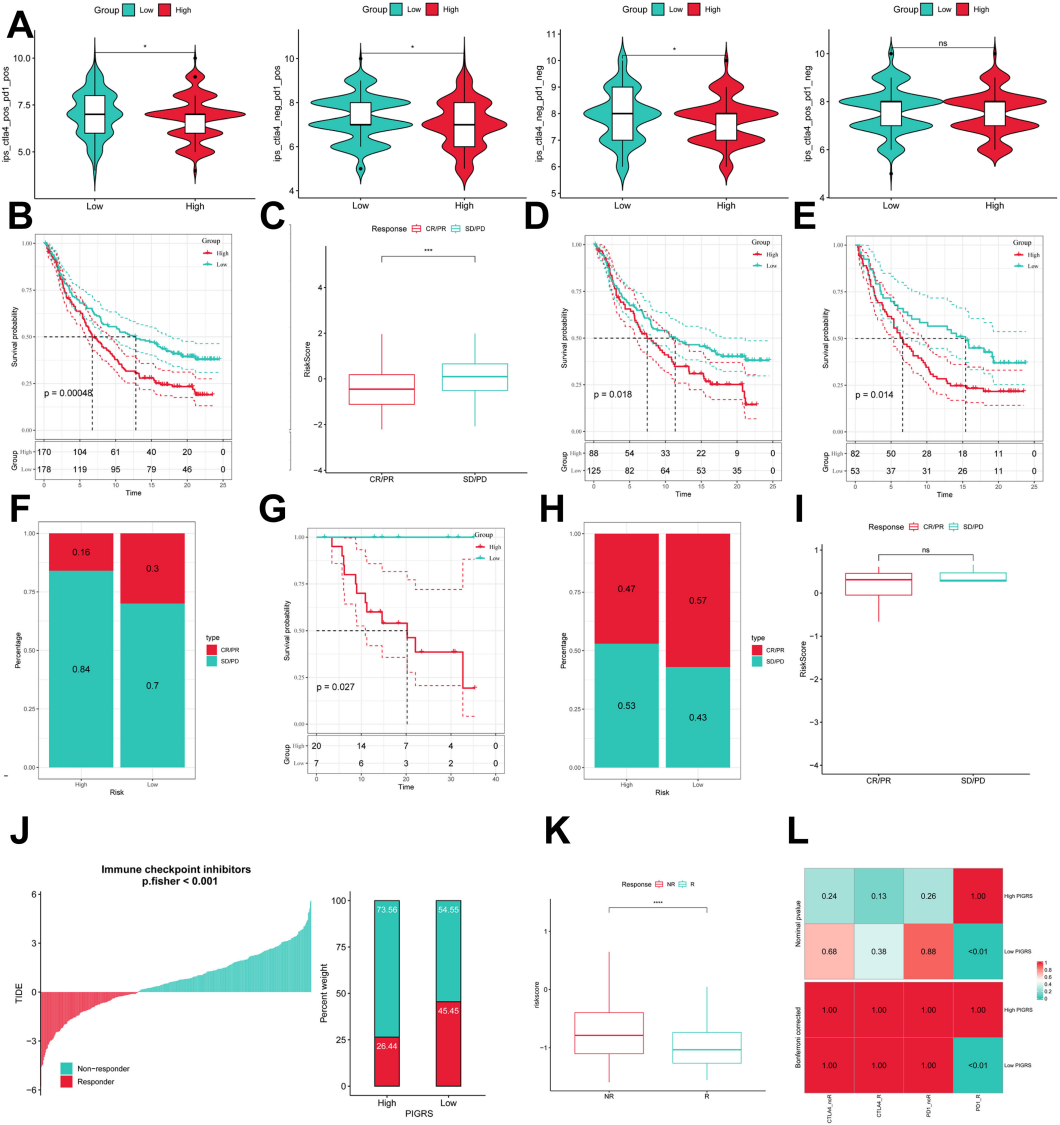
Predicting and validating the immunologic efficacy of PIGRS. **(A)** IPS scores of high and low PIGRS groups; **(B)** Survival curves of high and low PIGRS groups in the IMvigor210 cohort. **(C)** Box line plot depicting the difference in risk scores between CR/PR patients and SD/PD patients in the IMvigor210 cohort. **(D, E)** km curves for the high and low PIGRS groups in the IMvigor210 staging. **(F)** Proportion of CR/PR or SD/PD patients receiving immunotherapy in the high and low risk groups of the IMvigor210 cohort. **(G)** Survival curves for high and low PIGRS in the GSE78220 cohort. **(H)** Proportion of patients with CR/PR or SD/PD who received immunotherapy in the high and low PIGRS groups in the GSE78220 cohort. **(I)** Box line plot depicting the difference in risk scores between CR/PR patients and SD/PD patients in the GSE78220 cohort. **(J)** The TIDE algorithm predicts response to immunotherapy between the high and low ERGRS groups. **(K)** Proportion of R or NR patients receiving immunotherapy in the high and low PIGRS groups of the TCGA-LUAD cohort. **(L)** Submap algorithm predicting response to immunotherapy between the high and low PIGRS groups. ns, p >0.05,*p < 0.05,***p<0.001,****p < 0.0001.

To comprehensively assess the role of PIGRS in LUAD immunotherapy, we analyzed IPS scores obtained from the TCIA database. Higher IPS scores predicted a better response to ICI therapy, including PD-1 inhibitor and CTLA4 inhibitor therapy, and were classified into four categories: ips_ctla4_pos_pd1_pos, ips_ctla4_pos _pd1_neg, ips_ctla4_neg_pd1_pos, and ips_ctla4_neg_pd1_neg. Our results showed that all four categories were significantly elevated in the low-PIGRS group, suggesting that patients in the low-PIGRS group responded better to anti-CTLA4 therapy and the combination of anti-pd -1 and anti-CTLA4 than did the high-PIGRS group of patients (**Figure 10A**). In addition, we analyzed a cohort of uroepithelial cancers treated with anti-PD-L1 therapy (IMvigor210), and the low-PIGRS group had a significant survival advantage compared with the high-PIGRS group (**Figure 10B**). Also, patients in the low PIGRS group were more sensitive to immunotherapy (**Figures 10C, F**). In addition, stronger predictive ability was demonstrated in Stage I & II and Stage III & IV patients (**Figures 10D, E**). Next, in the GSE78220 cohort, low PIGRS also had a stronger ability to predict prognosis and immunotherapy benefit (**Figures 10G-I**). The Tumor Immune Dysfunction and Exclusion (TIDE) algorithm was used to assess patient response to immunotherapy and showed better responsiveness in the low PIGRS group (P [Fisher’s exact test] < 0.0001; **Figures 10J, K**). The Submap algorithm was performed on another group of melanoma patients receiving immunotherapy (GSE91061), and the results also showed a better response to PD-1 therapy in the low PIGRS (Bonferroni corrected p < 0.01, **Figure 10L**). These results demonstrate that the low PIGRS group is more likely to receive immunotherapy.

### Single-cell validation of PIGRS markers

**Figure 11:**
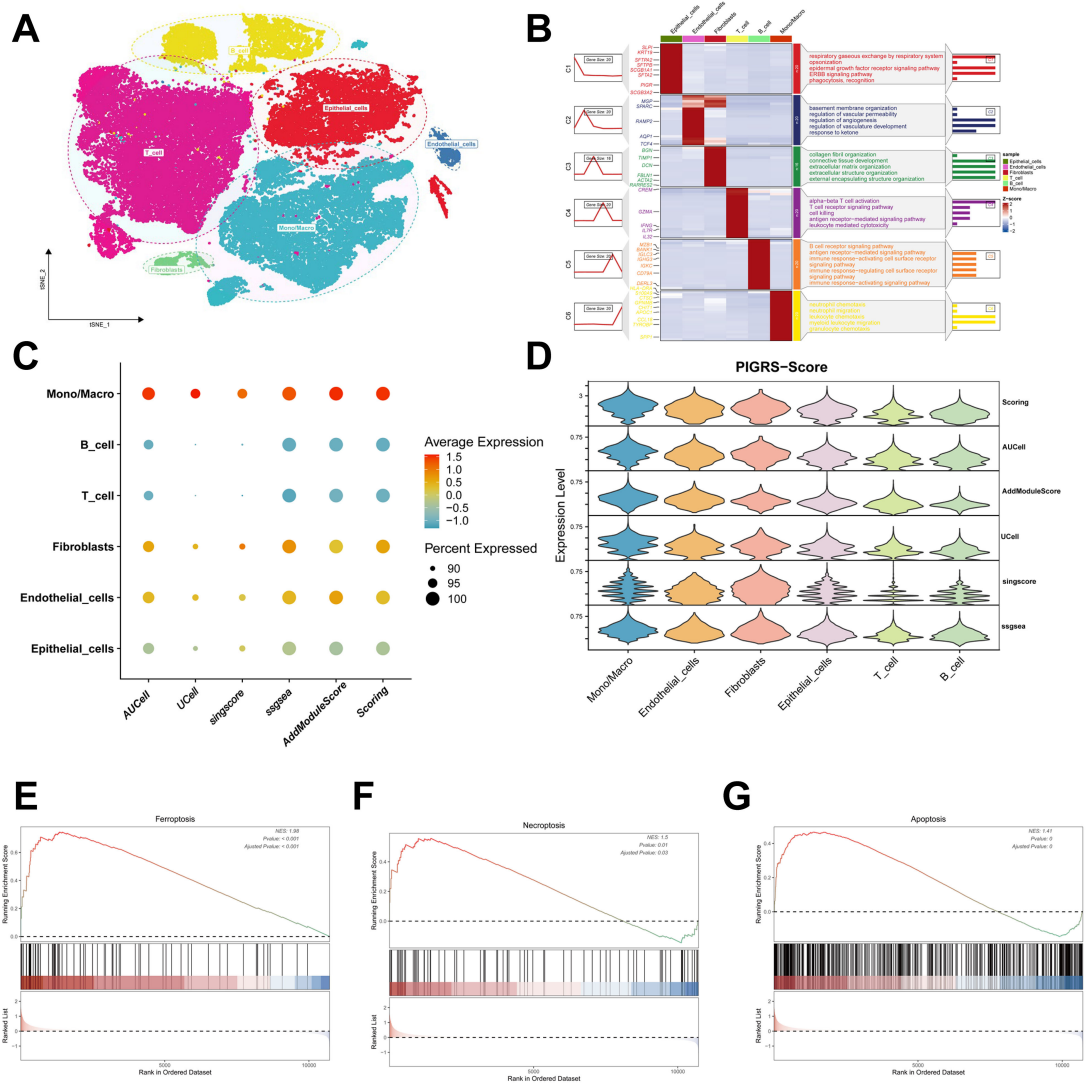
Validation of PIGRS markers. **(A)** t-SNE plot showing the cell types identified by marker genes. **(B)** Heatmap showing the 5 most important marker genes in each cell cluster. **(C, D)** Bubble map **(C)** and violin map **(D)** showing the enrichment scores of the PIGRS gene set for each cell type using AUCell, UCell, singscore, ssGSEA, and AddModulescore scores for the enrichment of the PIGRS gene set for each cell type. **(E-G)** Enrichment analysis of GSEA in the high PIGRS.Score group including Ferroptosis pathway **(E)**, Necrosis pathway **(F)**, Apoptosis pathway **(G)**.

We collected single-cell RNA sequencing data from 10 LUAD patients using the single-cell sequencing data in Bischoff et al. Using marker genes for different cell types, we labeled cells into 6 major clusters, namely T cells, fibroblast cells, macrophages/monocytes, endothelial cells, epithelial cells, NK cells, and B cells (**Figure 11A**). Enrichment heatmaps showed the marker genes and pathways enriched to for each cell population (**Figure 11B**). The distribution of the 15 genes in different cell types was demonstrated with violin plots and feature plots (**Supplementary Figures 5A–C**). We further used the AUCell, UCell, AddModuleScore, singsore, and ssGSEA algorithms at the scRNA-seq level to to quantify PIGRS scores at the scRNA-seq level. All algorithms showed that PIGRS scores were higher in macrophages, fibroblasts and lower in T and B cells (**Figures 11C, D**). Based on the PIGRS scores activity, we divided the cells into high and low PIGRS scores groups and identified differentially expressed genes (DEGs) between the two groups for GSEA enrichment analysis. The results showed that various cell death-related pathways were enriched in the high PIGRS scores group (**Figures 11E–G**). These results validated the markers of PIGRS.

### PSME3 promotes lung adenocarcinoma progression

**Figure 12:**
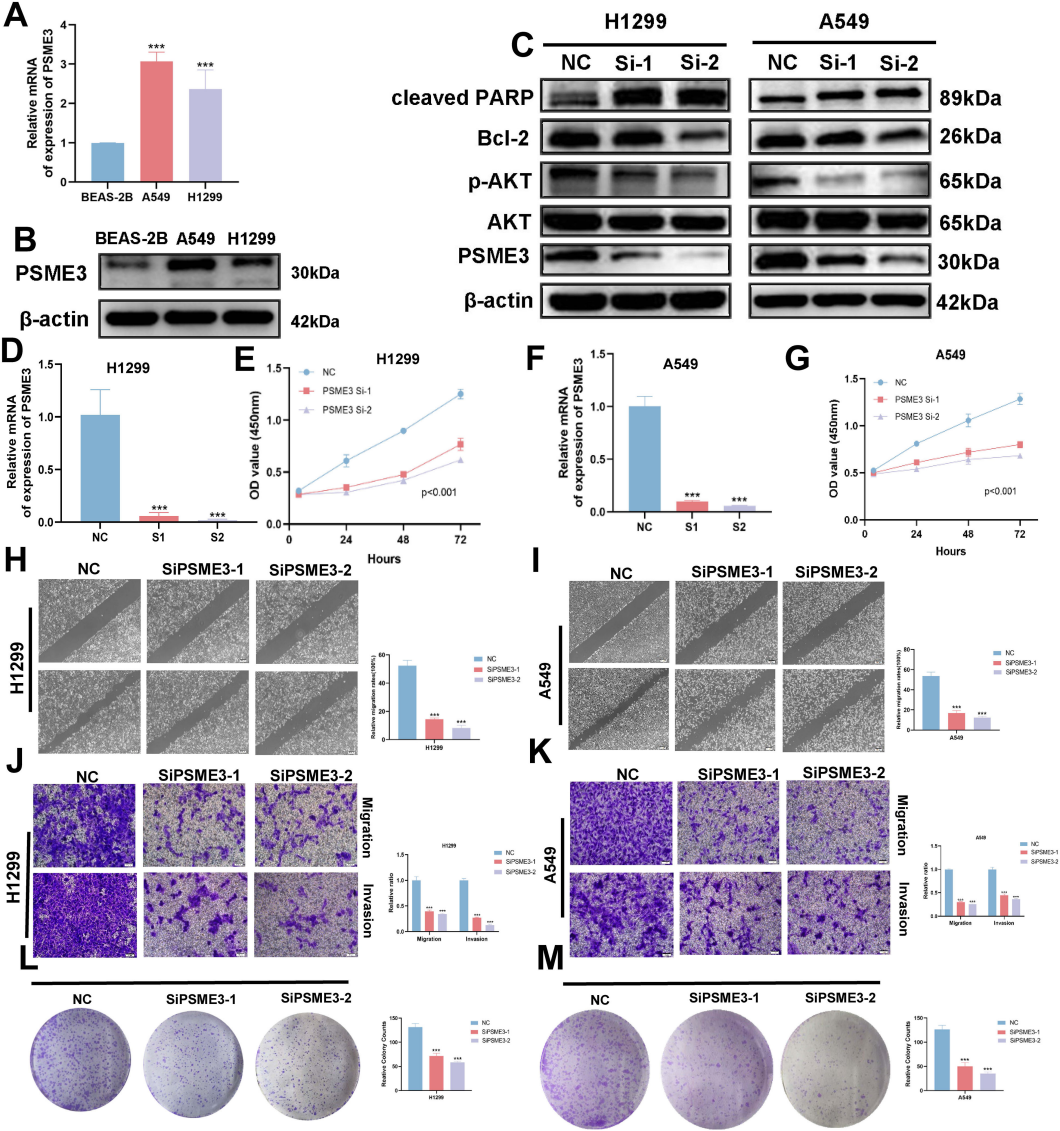
PSME3 affects the biological behavior of LUAD cells *in vitro*. **(A)** RT-qPCR to detect the expression of PSME3 mRNA. **(B)** The expression of PSME3 protein was detected by Western Blot. **(C)** Detection of β-actin, PSME3, AKT, p-AKT (Ser473), cleaved PARP, and Bcl-2 in PSME3 knockdown-treated A549 and H1299 cells by Western Blot. **(D, F)** RT-qPCR to detect the efficiency of Si-PSME3 transfection. **(E, G)** Growth curves of PSME3 knockdown-treated A549 and H1299 cells were determined using CCK8. **(H, I)** Cell scratch assay to detect the invasive ability of A549 and H1299 cells after PSME3 knockdown treatment. **(J, K)** Transwell assay to detect the invasion ability of A549 and H1299 cells after PSME3 knockdown treatment. **(L, M)** Colony formation assay was performed to detect the proliferation of A549 and H1299 cells. (“***” indicates p < 0.001).

In the Lasso + GBM algorithm in 101 Machine Learning, PSME3 is among the 15 most important genes and has not been fully investigated in LUAD. Therefore, we followed up with a series of cellular experiments on PSME3 in lung adenocarcinoma. RT-qPCR and Western Blot results showed that PSME3 expression of mRNA and protein in BEAS-2B was lower than that in A549 and H1299 cells (**Figures 12A, B**). To investigate the specific mechanism by which PSME3 affects lung adenocarcinoma, we detected that p-AKT was down-regulated after knockdown of PSME3 by Western Blot assay, suggesting that PSME3 is involved in the regulation of these pathways. And Bcl-2 was down-regulated and cleaved PARP was up-regulated after knocking down PSME3, suggesting that PSME3 may affect the apoptotic process of lung adenocarcinoma through the PI3K/AKT pathway (**Figure 12C**). In A549 and H1299 cell lines, siPSME3-1 and siPSME3-2 were selected for further study because of their transfection efficiency of more than 70% (**Figures 12D, F**). Cell proliferation was detected by CCK8 assay, and knockdown of PSME3 significantly inhibited proliferation of A549 and H1299 cells (**Figures 12E, G**). The results of cell scratch assay showed that knockdown of PSME3 significantly inhibited the migratory ability of A549 and H1299 cells (**Figures 12H, I**). The results of Transwell assay detected the migratory and invasive ability of cells, which was significantly decreased after knockdown of PSME3 (**Figures 12J, K**). The results of colony-formation assay showed that the proliferation ability of cells was significantly inhibited after knocking down PSME3 (**Figures 12L, M**). In conclusion, knockdown of PSME3 significantly inhibited cell proliferation, migration and invasion, and promoted apoptosis of lung adenocarcinoma cells. In terms of immunity, PSME3 shows a significant positive correlation with some immune checkpoints (**Supplementary Figure 6A**).

## Discussion

Despite significant efforts to develop comprehensive treatment strategies, the prognosis for patients with LUAD remains poor, with a 5-year survival rate of 15 percent (8). Exploring potential mechanisms and prognostic biomarkers may help precision medicine for cancer patients. Further discovery of potential mechanisms of tumor progression could lead to the development of new therapeutic strategies for lung adenocarcinoma.

In this study, we first collected 20 cell death and immunity-related genes. Then, we successfully established three molecular subtypes using ten clustering algorithms, which were associated with cell death, immunity, DNA methylation and somatic mutations. And among these three subtypes, patients in the CS3 group had a better prognosis than those in the CS1 and CS2 groups. Consistent results were also obtained in independent external datasets. In terms of gene mutations, the frequency of gene mutations was higher in the CS1 and CS2 groups than in the CS3 group, which may explain the poorer prognosis of the CS1 and CS2 groups.The frequency of TP53 gene mutations was higher in the CS2 group than in the CS1 group, whereas the frequency of TTN gene mutations was the highest in the CS1 group. TTNs are frequently detected in solid tumors with a high mutation rate and are associated with responsiveness to checkpoint blockade in solid tumors (61). TTN mutations may be a potential predictive biomarker for LUAD patients treated with ICIs (62). TP53 mutations are considered to be the most common gene-specific changes in human cancers and occur in almost all types of human tumors (63–65). TP53 plays a critical role in the control of cell cycle progression, senescence, DNA repair and aging, cell death and cell metabolism (66–68). The TMB of CS1 group was higher than the other groups, while the TMB of CS3 group was lower than the other groups. Exploring the functional differences among the three subtypes by GO enrichment analysis, we found that CS3 was mainly associated with immune-related pathways. In addition, considering that chemotherapy is the standard treatment for lung cancer, we estimated the chemosensitivity of each sample based on the IC50 value. The results showed that patients in the CS2 group were most sensitive to Sorafenib and Paclitaxel, while patients in the CS1 group were most sensitive to Cisplatin.

Machine learning algorithms are now widely used with clinical prediction (69). In this study, 20 cell death and immune-related genes were comprehensively analyzed for the first time. And by integrating machine learning techniques, a novel model, namely PIGRS, was developed. Multiple independent cohorts demonstrated that PIGRS has significant prognostic value and its predictive ability is higher than that of the previously published LUAD prognostic model. Our results suggest that PIGRS can be a valuable tool for guiding treatment decisions and improving patient prognosis.

By enrichment analysis, we found significant correlations between the high PIGRS group and DNA replication, cell cycle, and other proliferation-related biological processes, whereas the low PIGRS group was strongly associated mainly with some immune-related pathways. These findings provide partial insight into the more unfavorable prognosis observed in the high PIGRS group.

Our study revealed significant differences in somatic mutations between high and low PIGRS cohorts. Interestingly, the high PIGRS group exhibited significantly higher TMB, which has emerged as a novel prognostic biomarker strongly associated with immunotherapy response. We established a correlation between TMB and PIGRS and found a potential link between mutational load and immunotherapy response, providing a new perspective on checkpoint blockade therapy. In addition, our analysis showed that the high PIGRS group exhibited intra-tumor heterogeneity scores and that there was a positive correlation between PIGRS and MATH scores. Furthermore, our analysis showed that the low PIGRS group exhibited increased immune cell infiltration and higher expression levels of immunomodulators, including classical immune checkpoint molecules. However, patients in the low PIGRS group exhibited significantly higher levels of StromalScore, ImmuneScore, ESTIMATEScore and lower tumour purity compared to patients in the high PIGRS group. This suggests a complex interaction between PIGRS and the tumor immune microenvironment. IPS data downloaded from TCIA can provide predictive scores for evaluating a patient’s response to immunotherapy (70, 71). Higher IPS in the low PIGRS group suggests that patients with low PIGRS may have a more favorable response to ICI therapy. This study demonstrates the potential relevance of PIGRS in assessing immunotherapy response. And by SubMap algorithm results showed that the low PIGRS group had a higher response to immunotherapy. For patients with advanced LUAD, systemic therapy is the only option to improve survival. In addition to the use of immunotherapy-related drugs, we also tend to use some chemotherapeutic drugs, of which in the vast majority of cases, the low PIGRS group will have better therapeutic effects than the high PIGRS group, thus improving the survival time of LUAD patients.TIDE results also proved this point.

PSME3, also known as Proteasome Activator Complex Subunit 3, plays a crucial role in regulating essential cellular processes. In pancreatic cancer, PSME3 targets the cellular myeloid tumor (C-Myc) gene to stimulate lactate secretion. In pancreatic cancer, PSME3 targets the cellular myeloma oncogene (c-Myc) to stimulate lactate secretion (72). The Wang and others report PSME3 promotes lung adenocarcinoma development by regulating the TGF-β/SMAD signaling pathway (73). Dong and colleagues made a report Comprehensive analysis of PSME3: from pan-cancer analysis to experimental validation (74). In this study, we verified that interfering with PSME3 resulted in decreased proliferative capacity of LUAD cells by CCK8, Transwell and clonal origin assays, and verified that PSME3 may affect apoptosis of lung adenocarcinoma cells through the PI3K/AKT/Bcl-2 signaling pathway by Western Blot, and found that PSME3 was associated with the bioinformatic immune checkpoints were significantly positively correlated, suggesting that PSME3 may be a novel target in the immunotherapy of lung adenocarcinoma.

Overall, our findings suggest that PIGRS may serve as a valuable biomarker for predicting genomic patterns and immunotherapeutic responses in LUAD patients. Admittedly, limitations of our work remain. The current results highlight the need for prospective clinical trials to further validate the clinical applicability of our PIGRS. The mechanisms underlying the poor prognosis of patients at high PIGRS risk that we obtained should be further explored in wet trials. In addition, further animal experiments are needed to explore the functional role of PSME3 in lung adenocarcinoma, which could help to provide stronger clues to guide clinical application.

## Data Availability

The original contributions presented in the study are included in the article/**Supplementary Material**. Further inquiries can be directed to the corresponding authors.
