# Supplementary material for: Integrating multi-omics and machine learning survival frameworks to build a prognostic model based on immune function and cell death patterns in a lung adenocarcinoma cohort
Source: Front Immunol. 2024 Sep 13;15:1460547. doi: 10.3389/fimmu.2024.1460547 (PMC11427295; doi:10.3389/fimmu.2024.1460547)
Supplement: Supplementary file 2 [file DataSheet1.docx]

Supplementary Material





**Supplementary Figure.1**

Determining the classification of lung adenocarcinoma

(A) CPI and gap statistics were calculated to determine the optimal number of clusters for LUAD; (B) Ten clustering algorithms demonstrated; (C) Evaluation of sample homogeneity through silhouette scores derived from consensus ensemble results；(D) Heatmap of upregulated biomarkers in subgroups.


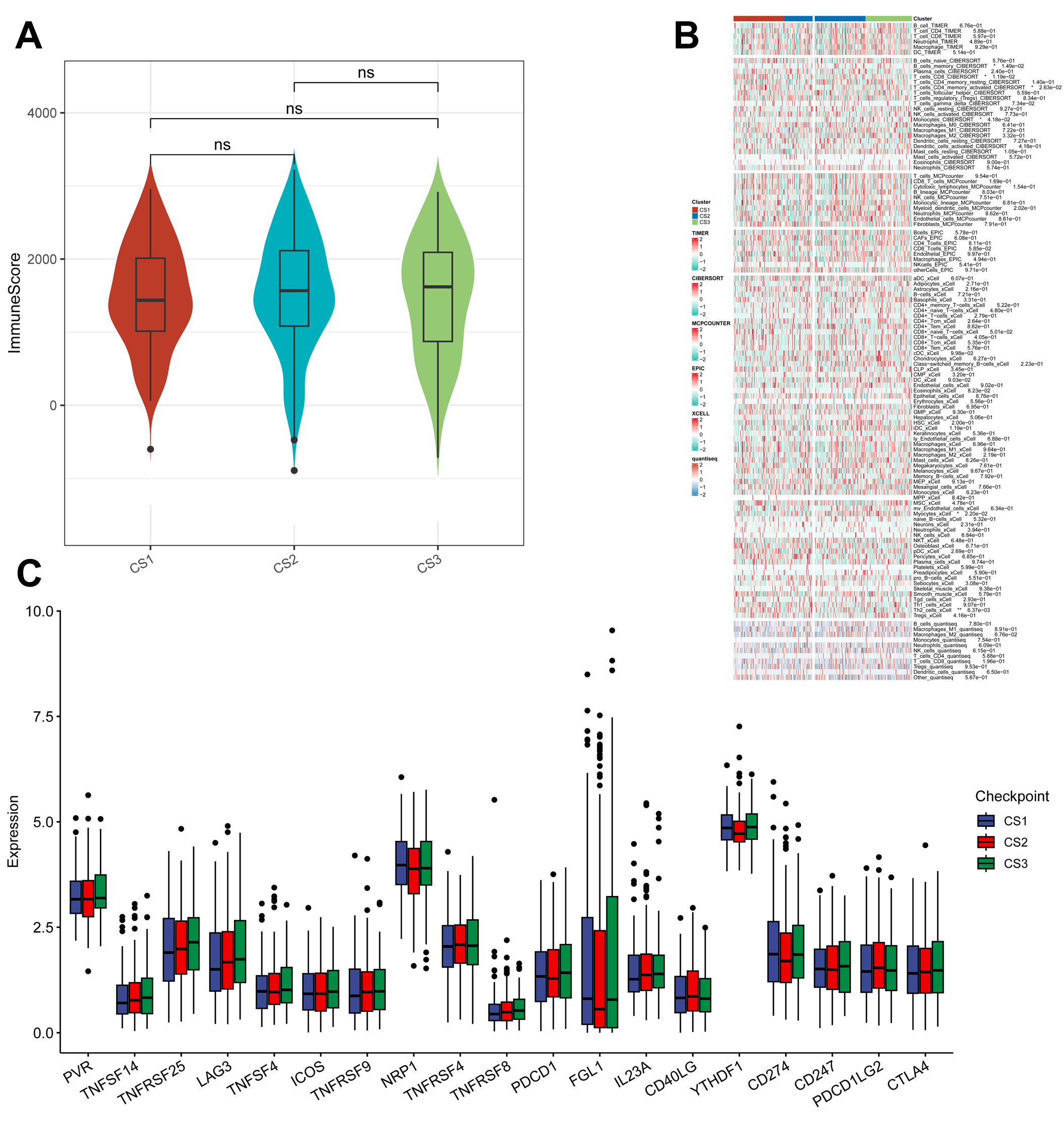


**Supplementary Figure.2**

Immune infiltration of different subtypes

(A) Box line plot showing immunity scores of different subtypes; (B) Heatmap showing different subtypes of immune cell infiltration；(C) Boxplots illustrating the expression of immune checkpoints in different isoforms.





**Supplementary Figure.3**

Inter-subtype treatment

(A) Response to Nivolumab in three subtypes based on the CheckMate immunotherapy cohort; (B) Assessment of response to Nivolumab in three subtypes；(C) Evaluation of three subtypes of response to immune checkpoint therapy. NCB，Non clinical benefit； CB，Clinical Benefit；R, Response；NR, Non Response.


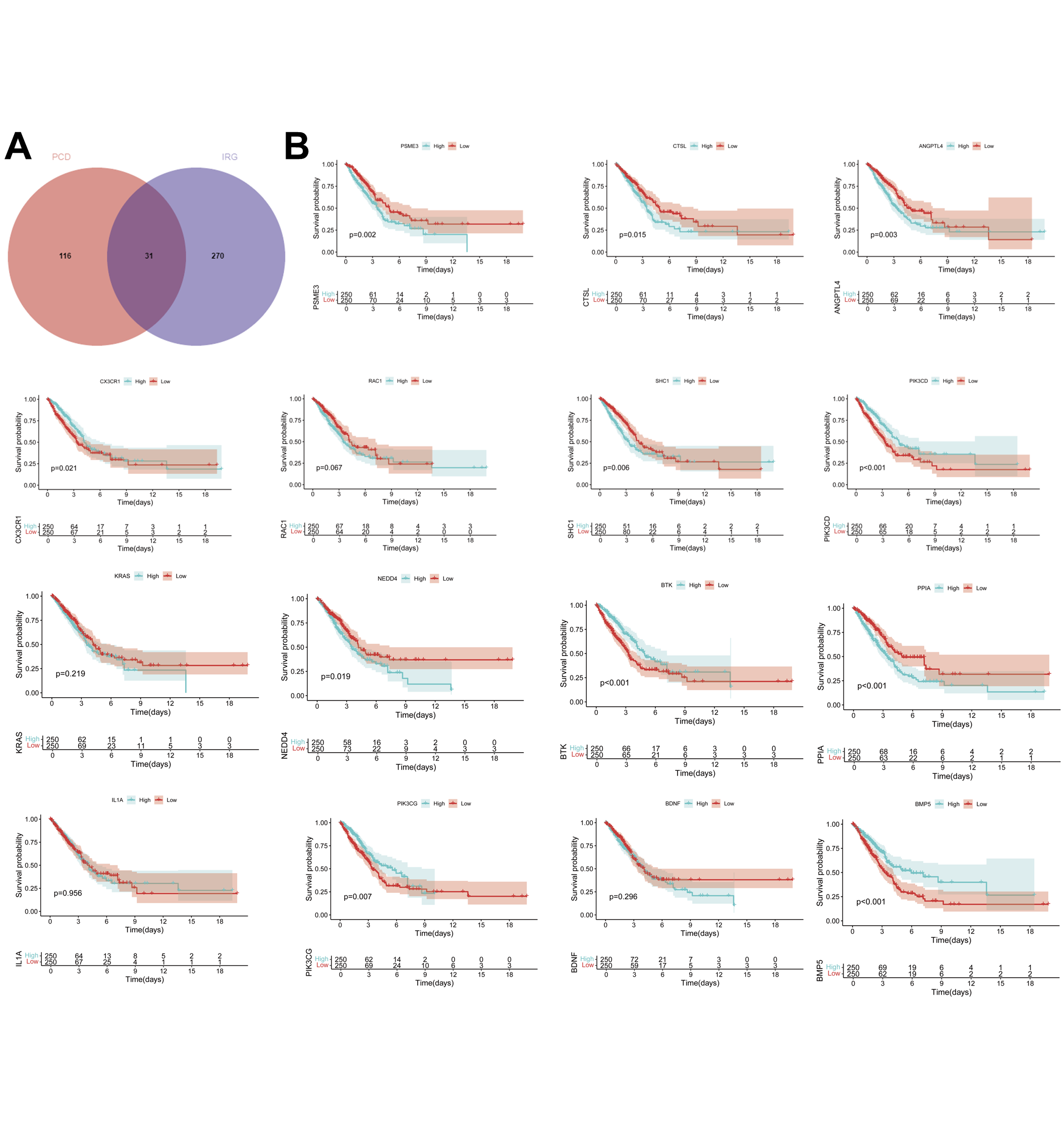


**Supplementary Figure.4**

PIGRS gene characterisation

(A) Venn diagram showing the intersection of PCD genes with IRG genes；(B) km curves for 15 PIGRS genes. PCD, Program cell death; IRG, Immune related gene.


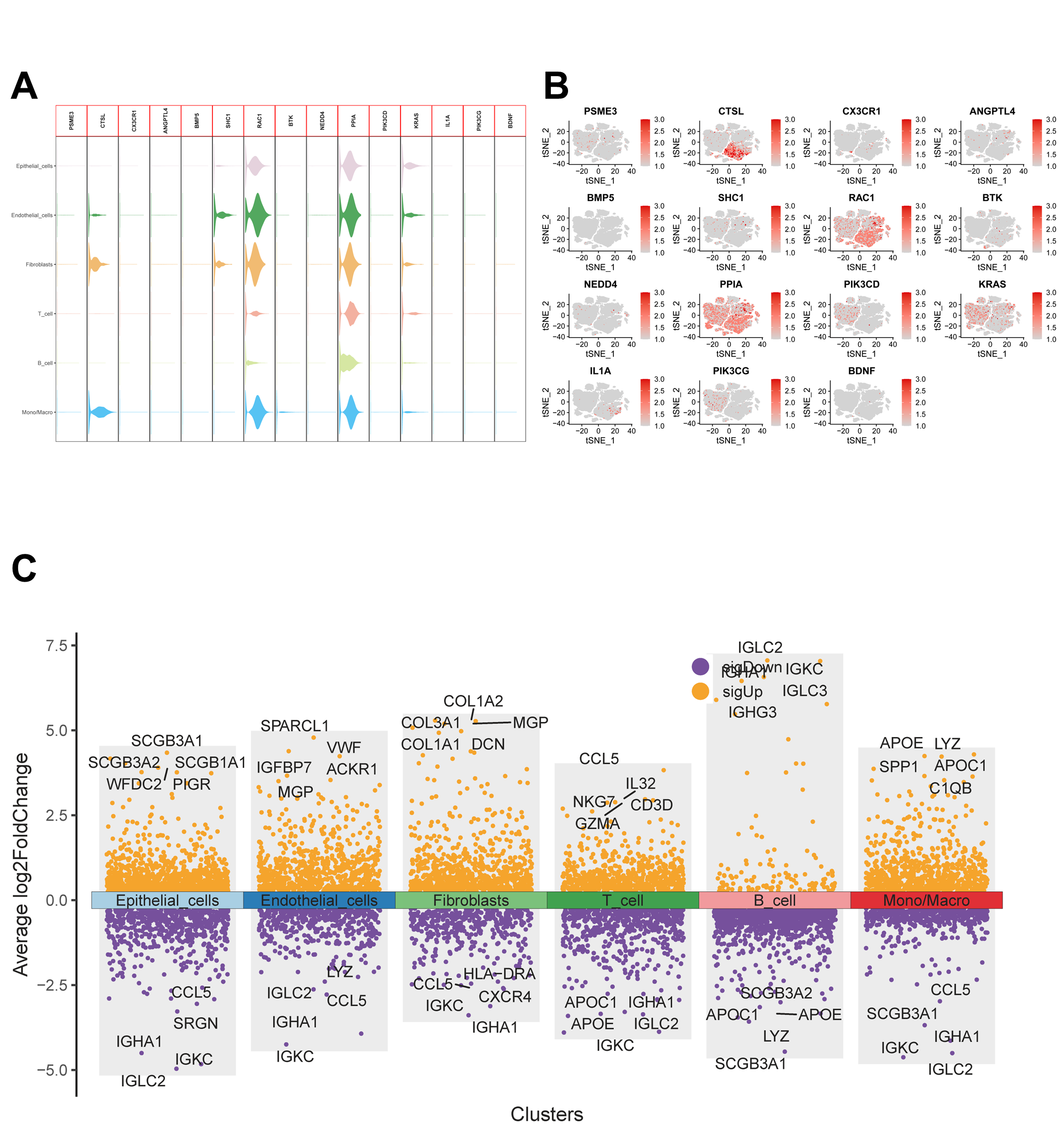


**Supplementary Figure.5**

Single cell demonstration of PIGRS gene signature

(A) Violin expression of 15 PIGRS genes；(B) feature plot of 15 PIGRS genes showing expression；(C) Single-cell volcano diagram.


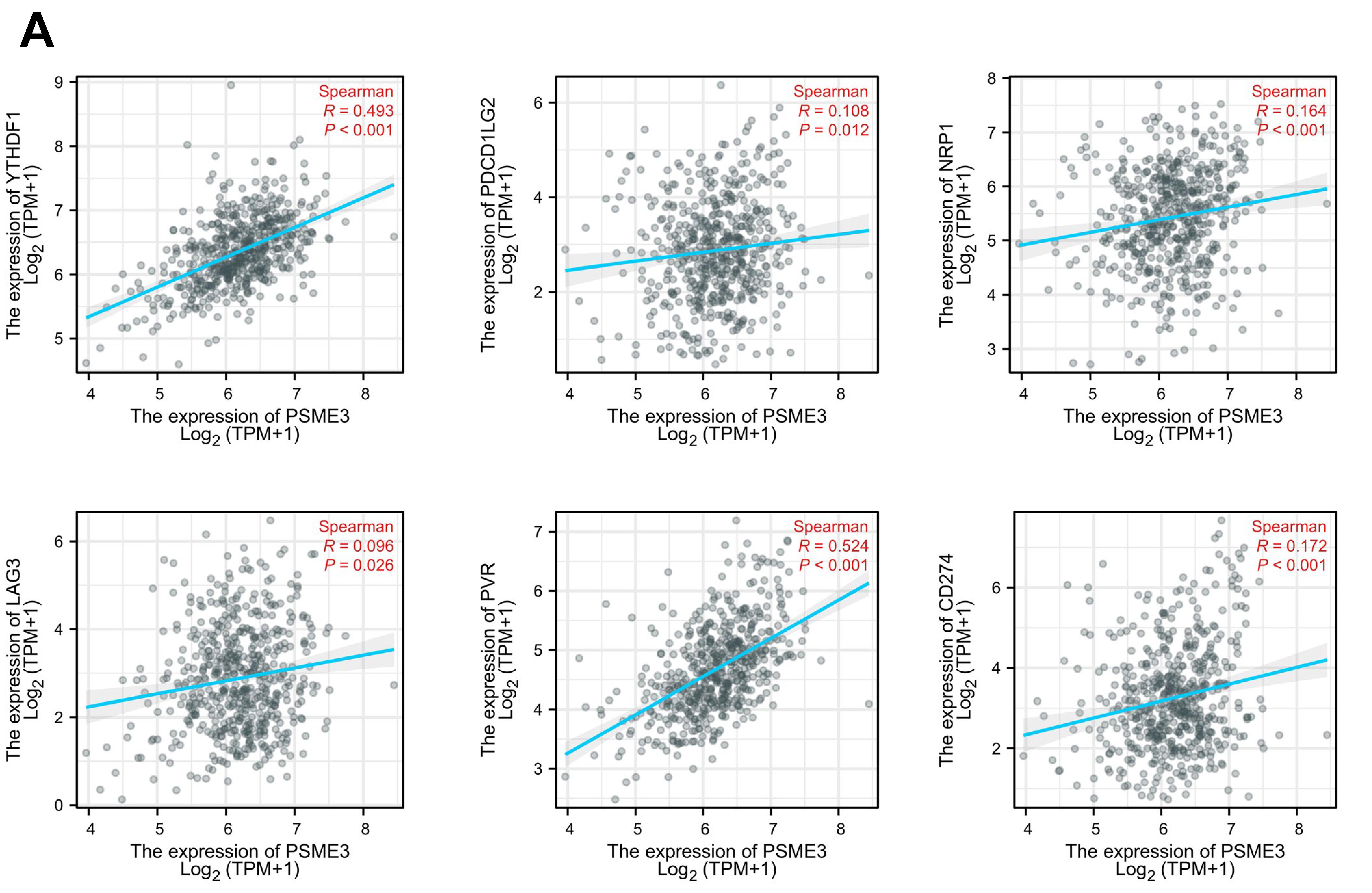


**Supplementary Figure.6**

Correlation between PSME3 and immune checkpoints
